# Observations on Yellow Fever, with Special Reference to Diagnosis, Prognosis and Treatment

**Published:** 1888-09

**Authors:** John P. Wall

**Affiliations:** Tampa, Fla.


### OBSERVATIONS ON YELLOW FEVER, WITH SPECIAL REFERENCE TO DIAGNOSIS, PROGNOSIS AND TREATMENT.

BY JOHN P. WALL, M. D., TAMPA, FLA.

The following remarks on yellow fever are based on clinical observations during the epidemic of that disease last fall in Tampa :

The etiology of this fever is but little better, if any, understood than its pathology, and in these two points are to be found the individual characteristics, so to speak, of the disease. The idea of yellow fever being only a more malignant grade of malarial fever can only be entertained by very superficial observers, or those who have never passed through an epidemic of this fever. Notwithstanding this broad assertion, it is not by any means easy, nor in fact always possible, to determine whether any single case is yellow fever or not, and this is especially the case preceding the inception of an epidemic. To arrive at a conclusion on this point, quite a number of circumstances in connection with the case, apart from the symptoms, have to be taken into consideration and then, reasoning by a process of exclusion, we may be able to approximate at least the probable truth. The recent history of the patient as to being exposed to infection in another place, on board ship or otherwise, and whether or not the patient has had a previous attack of this specific fever, are circumstances of the first importance to be taken into consideration. Though the patient may be from an infected place, or has been otherwise exposed to the infection, yet if it can be positively established that he has had yellow fever, the probability of his having a second attack is so unlikely and remote as to warrant the exclusion of the yellow fever hypothesis in arriving at a diagnosis. But where the patient has recently been exposed to infection and possesses no immunity acquired by a previous attack from this specific fever—in other words, is not acclimated—then in such a case there is a probability of its being yellow fever, and advantage should be taken of the doubt and the case managed as a case of yellow fever without waiting for a positive diagnosis. The management of such a case consists in the complete and thorough isolation of patient and attendants, and on the termination of the case the prompt institution of measures for efficient disinfection of everything, even to premises, that might have been contaminated.

In its symptomatology yellow fever presents no well-marked indications of its true character in the beginning, nor for that matter at any time, in mild cases, during its progress. Its attack is generally ushered in by a chill or chilly sensation, as in most other fevers and the pyrexiæ. With the rise of fever there is generally intense frontal headache with pain in the small of the back and extremities, especially the legs. A feeling of tiredness in the legs, with a sense of general prostration, is usually experienced. Nausea from the beginning is not an uncommon symptom, though not always present. Incessant nausea, with much retching or vomiting during the first fever, indicates the case to be of a severe type. The tongue is generally coated with white or whitish fur, is sometimes enlarged and almost invariably presents red edges on the sides. The temperature for the first three days may range in different cases from 102 to 106 degrees F., with a slight morning remission in the majority of cases. The pulse for the first three days may range in different cases from 90 to 110 per minute, though not so frequently above as below 100 beats per minute.

The skin is generally burning hot, rarely dry, and not unfrequently bathed in profuse perspiration. In many cases after the fever sets in, the extremities remain cool, while the body heat is intense and pungent. And it is in this class of cases where mustard pediluvia or mustard sitz-baths have been found so useful and beneficial in restoring the equilibrium of the circulation, as to render the mustard bath an almost universally common remedy in the beginning of treatment. In severe cases albumen will be found in the urine on the *third* day, as a rule. Its presence is always a grave symptom, but as it is rarely—never in my observation—to be detected before the third day, it is impossible to determine up to that time whether the case is to be of a mild or grave type. In all cases where albumen is not present in the urine on the third day or at the expiration of seventy-two hours from the inception of the fever, the case will, as a rule, prove to be of a mild type, and the patient and friends can be assured of a speedy recovery if great prudence in eating and drinking is exercised for two or three days more.

And right here it is well to distinctly emphasize the fact that it is the acute parenchymatous nephritis, as indicated by the presence of albumen in the urine on the third day, which is the most marked characteristic of the graver type of this fever, and without which the icteric hue of the conjunctivæ and skin is rarely, if ever, developed. But it must be borne in mind that by no means do all cases of this fever, as it presents itself in this country, have albumen in the urine. And it is the presence of albumen in the urine on the third day, subsequently followed by the icteric hue of the conjunctivæ and skin, by which this fever can be positively diagnosticated. These two symptoms—albumen in the urine and the icteric hue—at the proper time and in proper sequence, render the diagnosis of yellow fever as positive from a scientific point of view, as it is possible to diagnosticate any other disease by physical symptoms.

The later writers and authors speak of albumen appearing in the urine in yellow fever, but fail to indicate at what time or stage of the disease the albumen is to be found, and they also fail to say that it is not present in all cases—two facts of the first importance. In the *London Lancet*, of the 17th of March of this year, Dr. Robert Lawson, Inspector-General of the British army, who has served in Jamaica, says: "On the evening of the third day, or morning of the fourth, the urine generally presents traces of albumen," thus confirming my observation as to the time when the presence of albumen in the urine may be expected. He fails to state, however, that albumen is not present in all cases of this fever, a fact of the highest importance in eliminating much of the confusion in regard to the diagnosis. In mild cases, where no albumen is found in the urine on the third day, the fever will terminate on the expiration of seventy-two hours, sometimes earlier, not to return and the patient will progress to rapid convalescence without farther trouble.

Yellow fever is generally described by authors as a fever of one paroxysm of seventy-two hours' duration, followed by a stage of apyretic calm of variable length from one to twelve hours, and this is succeeded by the recurrence of fever of more or less intensity, accompanied with nausea and vomiting of white glairy mucus at first, then flecked with dark specks and shreds which have been compared to bees' or butterflies' wings, finally terminating in the coffee-grounds-like black vomit. Now, all of this is true of typically bad cases, as a rule; but this secondary fever and nausea are very rarely to be met with in mild cases devoid of albumen in the urine on the third day. And in all cases where there are no nephritic complications, as indicated by the absence of albumen in the urine, yellow fever ends on the third day, or perhaps earlier, and convalescence practically sets in and recovery is soon complete.

Thus it will be seen that practically there are two types or grades of this fever to be met with in an epidemic—one whose main feature is an acute parenchymatous nephritic complication developed by the third day and the other, having no such complication, ends with the first paroxysm of the fever and almost invariably terminates in speedy recovery, the patient being up and about as early as the fifth or sixth day. Not so, however, where albumen is detected in the urine on the third day; for while in this class of cases the high fever of the first three days may subside, and the pulse rate return to the normal, or even fall much lower, and the temperature be anywhere from 97 to 104 degrees F., the patient still continues in a most critical condition and not unfrequently so remains for several days or even longer. In some instances the temperature and pulse are normal, and so remain after the subsidence of the first paroxysm of the fever; but there are plenty of indications, and especially the albumen in the urine, to show the serious condition of the patient. It is doubtless this class of cases to which relapses are attributed by a certain class of physicians who are always trying to explain away why they failed to cure all their cases of yellow fever.

The amount of albumen in the urine varies in different cases, ranging from five to seventy-five per cent. of volume of the total amount; and, as a rule, the greater the amount of albumen the more serious and less hopeful may be considered the prognosis. In these nephritic cases the eyes begin to show the icteric hue on the fifth day, and on the sixth or seventh day, the skin begins to turn yellow. In rapidly fatal cases, terminating as early as the fourth or fifth day, the yellow hue appears earlier and is always well marked after death. This yellow color, which is characteristic of this fever in its worst and most fatal type, and has given the fever its name, is very rarely, indeed, if ever, present in cases unaccompanied by the nephritic complication. This is repeated because it is a most important fact to remember.

Hemorrhages from the mouth and nose are not infrequent in the severe grade of cases having the nephritic complication. The urine may be tinged with blood, imparting to the coagulated albumen a dark color, but hemorrhage from the urinary organs is seldom profuse; and as these hemorrhages never occur in the first three days' paroxysm of the fever, and only in cases with nephritic complication, there should never be any trouble in differentiating yellow fever from hemorrhagic malarial fever. The eyes become blood-shotten in no inconsiderable number of cases. Epistaxis and bleeding from the gums are the more common forms of hemorrhage met with in this fever, and the nose-bleed is often alarming. Among females passed puberty, more or less uterine hemorrhage is almost certain to occur during the course of the fever, making its appearance from the third to the fifth day. Even in mild cases this uterine hemorrhage is rarely absent—and by mild cases it is understood that those without the nephritic complication are thus designated.

The icteric hue of conjunctivæ and skin has very much the appearance of ordinary jaundice, though it is hardly likely that it is produced by the same cause—retention of bile in the blood—for the reason that bile is not very frequently found in the urine, as is universally the case in ordinary jaundice. One of the tests for bile in the urine is to add albumen and precipitate it with heat and an acid, when the coagulated albumen will assume a greenish color imparted by the presence of biliverdin. In cases with the nephritic complication the albumen is already present in the urine, but it is only occasionally that the precipitat assumes the greenish color of the biliverdin. From the time the yellowness of the skin becomes fully developed until it begins to fade, the capillary circulation is extremely sluggish—so much so, in fact, that firm pressure on the chest with the open and separate fingers leaves their marks on the surface for a number of seconds, if not a full minute. Judging from the tendency to hemorrhage and the capillary stasis, which are such prominent features in severe cases of this fever, and taking into consideration the absence of bile in the urine in the large majority of cases, the conclusion is highly probable, if not positive, that this yellowness must be mainly dependent on pigmentation of the eyes and skin by the coloring matter of the blood instead of being occasioned by the retention of bile in the blood—as in ordinary jaundice—as is generally supposed and believed.

Suppression of urine, or a very scant secretion more or less tinged with blood, only occurs in the third stage of the fever—rarely, if ever, during the paroxysm of the first three days. And it is only in this graver type of cases with the nephritic complication that suppression of urine takes place, to be followed by spasmodic twitchings, stupor, convulsions and death. These muscular twitchings are especially noticeable about the face. These nervous phenomena are supposed to be the effects of uræmic poisoning as a result of non-excretion of urea by the kidneys and its retention in the blood. But be that as it may, their occurrence is almost a certain prelude to a fatal termination, and in a prognostic sense is as bad, if not worse, than black vomit. Hiccough is never met with in the first fever—*i. e.*, before the fourth or fifth day, and is a symptom of the most unfavorable import.

The injected and watery eyes, swollen face and anxious countenance, so generally depicted by writers on this fever, may be noticed in a large number of cases, probably the majority. The detection of albumen in the urine on the third day of the fever, and the subsequent icteric hue of the eyes and skin, establish the diagnosis of this fever beyond all doubt. But, as before pointed out, these two symptoms are absent in mild cases—those unattended with the nephritic complication—and hence the frequent disputes and contentions as to the diagnosis in the beginning of an epidemic. The duration of the paroxsym of the fever for only seventy-two hours, or even perhaps a shorter time, and its abrupt termination, without the use of quinine, should excite, however, grave suspicion of its character; and this suspicion will be greatly strengthened if the chill came on in the night, and especially the latter part of the latter half of the night. Dengue is the only other fever of our climate which may be ushered in by chill at night, but its symptoms are too well marked, and its being a proverbially non-fatal disease, to admit of its being mistaken for yellow fever, or, *vice versa*, yellow fever being mistaken for dengue, by any one but the merest tyro in the profession, especially after a few days' watching and observation. This brevity of the first paroxysm of yellow fever in mild cases, its subsidence without the use of quinine, the severe frontal headache with pain in the back and extremities and the absence of the second paroxysm and eruption on the extremities of dengue, will rarely fail to indicate to the experienced physician the true nature of the case. Many yellow fever patients emit a peculiar odor which the experienced physician has no difficulty in recognizing, though he may not be able to describe it by comparing it to any similar smell.

The matter of early diagnosis is a most important one in our Southern cities and towns, as it is generally conceded, and my own experience tends to confirm the fact, that if the first case or cases are recognized and proper measures of isolation and disinfecting are instituted, an epidemic may be prevented, especially in places whose atmosphere is not vitiated with miasmatic exhalations from accumulated filth. Hence the importance of ever being on the alert and carefully investigating any and every case of fever that comes under observation during the season when the fever usually prevails—the summer and fall of the year.

Daily observations should be made for albumen in the urine from the first to the fourth day, and if its presence is detected on the third day the diagnosis is probable, and if this is succeeded on the sixth or seventh day by the icteric hue of conjunctivæ and skin, the diagnosis is positive. If the case is mild, however, but with severe frontal headache and other symptoms already detailed, and abruptly terminates on the expiration of the third day, although no albumen may have been found in the urine, the diagnosis is probable of its being yellow fever; and the benefit of the doubt should be given to the community, and all necessary precautions should be taken to prevent the dissemination and spread of the infection. Of course the previous history of the patient as to the probability of his having been exposed to the infection, and as to his ever having had yellow fever before, should be duly investigated.

As to treatment, little need be said, as, unfortunately, so far no specific treatment has been found. The mild cases of the fever—those destitute of the nephritic complication—will get well spontaneously with or without treatment, as a rule. And it is probably owing to a failure to recognize the two types of this fever, which have been pointed out in this paper, that is to be found the reason why so many different remedies have been vaunted and then laid aside, and why so much has been said of different epidemics differing in their phase, and remedies found good in one proving futile and useless in another.

The main indications are to restore the equilibrium of the circulation by mustard pediluvia or baths, evacuate the bowels with some efficient, though mild purgative, and control the febrile excitement by the use of antipyretics—antipyrin and antifebrin. So far as my experience goes mercurials do not appear to possess any special advantage, except possibly that the stomach is not so likely to reject them as 'often as it does more bulky drugs. Doubtless the common, though possibly erroneous, prevailing belief that yellow fever is only an intensified form of bilious fever, and that the icteric hue is dependent on the same cause as that producing jaundice, and hence the fault is the liver, has always attached more importance to the use of mercury in the shape of calomel in this disease than experience justifies Castor oil or the more common salines will be found about as good evacuants of the *primæ viæ* as anything else. Quinine is a useless drug in yellow fever unless there is some evidence of a malarial complication, when it may be advantageously used during convalescence, but it is perfectly valueless in controlling the disease. Administered in mild cases which would naturally recover, quinine may, and doubtless often has received the credit of having cut short the fever as a *post hoc propter hoc* conclusion. Its being withheld, even in mild cases, is advantageous in a diagnostic point of view, for if the fever spontaneously ends by the end of seventy-two hours or earlier, the presumption is against it having been of malarial type.

Antipyrin in ten grain doses every three hours appears to have a very beneficial effect in reducing temperature, promoting diaphoresis and relieving headache and other pains. Its advantages over antifebrin are its analgesic properties and greater solubility, which renders its administration by enema in twenty or thirty grain doses almost as efficient as when administered *per orem.* And this method of administration becomes sometimes necessary on account of nausea and excessive irritability of the stomach. The use of opiates is always hazardous unless there was some way of determining anterior to the third day that no nephritic complication would be developed, as their use where this is the case is apt to induce suppression of urine.

Of course in mild cases, where there is no albumen in the urine, it is not certain that their use is hurtful, but the difficulty as to their use arises from our inability to determine this very important point as to the nephritic complication; and hence the safe rule is to eschew the use of opiates entirely in the treatment. An occasional exception to this rule may be made in cases complicated with other troubles and where the kidneys are not or only slightly affected.

As to food and drink, the less taken the better, so as to keep the stomach as quiet as possible. Cold drinks are decidedly bad in the great majority of cases. Hot drinks are borne much better, and hence, doubtless, the common practice of administering lemon leaf and other teas. Possibly the benefit derived from these hot drinks is dependent on their constringing effect on the capillary circulation of the mucous coat of the stomach, on the same principle that injections of hot water are used in gynecology. For nourishment a tablespoonful of equal parts of milk and lime water may be given every two hours, though if the patient is not naturally feeble, "Tannerism," or starvation may be practiced.

In the graver type of cases, with the nephritic trouble, no line of treatment, after the third day, promises any certainty of success. So long as the quantity of albumen in the urine remains comparatively small, and the kidneys continue to act tolerably freely, there is encouraging hope of the patient "pulling through," though there is no means of telling when or how soon the action of the kidneys will become fatally impeded or suppressed and the case terminate in stupor, convulsions and death, with or without black vomit toward the end. It is in this class of cases that we are constrained to recognize the impotency of our art and utter helplessness in the presence of this dread disease. In this latter stage the tincture of digitalis in ten to fifteen drop doses every two or three hours may prove of some advantage as a heart tonic and diuretic. Of course, alcohol preparations are indicated to sustain the flagging vital powers; but, unfortunately, like a great many other remedies, disappointment is only too frequently the result of their use. In the mild cases without the nephritic trouble any preparation of alcohol is rarely indicated, may be occasionally indicated in the nephritic cases where the quantity of albumen is small and the kidneys act fairly well, while in the worst grade of cases, it, like everything else in the way of remedies, is likely to prove of little avail.

No remedies proved specially useful in relieving nausea and hiccoughs. Cocaine was tried without benefit. Carbolic acid in small doses proved a failure. In fact, nothing but as complete abstention as possible from food and drinks appeared to do any good.

NOTE.—These observations are based upon one hundred and thirty cases that I have treated myself in which the urine was tested, besides seeing a large number in consultation where the urine was examined also. Dr. J. Y. Porter, who was here during the epidemic, and had charge of the fever hospital and the Government relief measures, admits that his observations tally in the main with mine.

---

PROTECTION.—The physicians of the United States—125,000 strong and through their wives and children representing at least 500,000—are as worthy of *protection* as either the wool-growers or manufacturers of surgical instruments. Did any one ever hear of *protecting* a physician? Our country is open to physicians from England, Ireland, Scotland, Canada, Germany, Japan and China. They are welcomed to our shores and, like foreign wines, they are considered far ahead of the native product! Let us arise in the dignity of Americans and demand that these foreign products be taxed (protection means tax). Do we not owe this to ourselves, our wives and our children? If protection is a good thing, let us all have some—there should be no discrimination—there should be no favored class. American artisans, musicians and painters are clamoring for their rights and have been listened to. Let us demand a heavy tax on the ready-made doctor—a manufactured commodity—that demands an entrance to our domain, and let the duties be *ad valorem*, specific and combined.—*Medical Times.*

